# DNA methylation profiling reveals novel pathway implicated in cardiovascular diseases of diabetes

**DOI:** 10.3389/fendo.2023.1108126

**Published:** 2023-02-15

**Authors:** Shengqing Hu, Lulu Chen, Tianshu Zeng, Wenyi Wang, Yan Yan, Kangli Qiu, Yajuan Xie, Yunfei Liao

**Affiliations:** ^1^ Department of Endocrinology, Union Hospital, Tongji Medical College, Huazhong University of Science and Technology, Wuhan, China; ^2^ Hubei Provincial Clinical Research Center for Diabetes and Metabolic Disorders, Wuhan, China

**Keywords:** diabetes, cardiovascular diseases, DNA methylation, methylated DNA immunoprecipitation, signaling pathway

## Abstract

**Objective:**

Epigenetics was reported to mediate the effects of environmental risk factors on disease pathogenesis. We intend to unleash the role of DNA methylation modification in the pathological process of cardiovascular diseases in diabetes.

**Methods:**

We screened differentially methylated genes by methylated DNA immunoprecipitation chip (MeDIP-chip) among the enrolled participants. In addition, methylation-specific PCR (MSP) and gene expression validation in peripheral blood of participants were utilized to validate the DNA microarray findings.

**Results:**

Several aberrantly methylated genes have been explored, including phospholipase C beta 1 (PLCB1), cam kinase I delta (CAMK1D), and dopamine receptor D5 (DRD5), which participated in the calcium signaling pathway. Meanwhile, vascular endothelial growth factor B (VEGFB), placental growth factor (PLGF), fatty acid transport protein 3 (FATP3), coagulation factor II, thrombin receptor (F2R), and fatty acid transport protein 4 (FATP4) which participated in vascular endothelial growth factor receptor (VEGFR) signaling pathway were also found. After MSP and gene expression validation in peripheral blood of participants, PLCB1, PLGF, FATP4, and VEGFB were corroborated.

**Conclusion:**

This study revealed that the hypomethylation of VEGFB, PLGF, PLCB1, and FATP4 might be the potential biomarkers. Besides, VEGFR signaling pathway regulated by DNA methylation might play a role in the cardiovascular diseases’ pathogenesis of diabetes.

## Introduction

The worldwide incidence of diabetes has increased sharply in recent years (1). It is acknowledged that cardiovascular diseases are the leading cause of morbidity and mortality among diabetic patients (2, 3). Compared to microvascular complications, macrovascular complications including cardiovascular diseases contribute more to mortality in diabetics (4). However, the underlying mechanism of diabetic cardiovascular diseases remains largely unknown. Meanwhile, the present interventions for cardiovascular diseases in diabetic patients show limited effectiveness. Thus, understanding the mechanisms of diabetes-associated cardiovascular diseases are of great importance.

The Diabetes Control and Complications Trial (DCCT) and its follow-up research (Epidemiology of Diabetes Interventions and Complications Study, EDIC) identified a widespread phenomenon known as “metabolic memory”. Several studies have implicated the epigenetic role of hyperglycemia in ‘metabolic memory’ (5–7). It has been demonstrated that hyperglycemia could regulate gene expression by epigenetic modifications, such as DNA methylation, which can persist after glucose normalization (8). Meanwhile, increasing evidence showed that epigenetic modifications might play a critical role in the pathophysiology of diabetes and its related cardiovascular complications (9, 10). Recently, DNA methylation is involved in the pathogenesis of diabetes and microvascular complications (11). However, the role of DNA methylation in macrovascular complications in diabetes remains uncertain. The whole genome was hypomethylation in peripheral blood mononuclear and aortas of 4-week-old ApoE-null mice, a classic atherosclerotic animal model, in which stage that none of histological signs of atherosclerosis appears (12). Furthermore, altered DNA methylation of several candidate genes linked to atherosclerosis was identified in vascular smooth muscle cells (VSMCs), endothelial cells (ECs) and mouse models (13, 14).

Moreover, risk factors for type 2 diabetes, such as obesity and aging, could also affect the methylome in non-diabetic subjects, which might trigger impaired insulin secretion, insulin resistance, and the progression of diabetes (15). As other risk factors for cardiovascular diseases, hyperhomocysteinemia and hypercholesterolemia have also been involved in DNA methylation association with candidate genes involved in atherosclerosis (14, 16, 17). Therefore, elucidating whether epigenetic alterations are involved in the development of diabetic macrovascular diseases would provide a potential therapeutic target for these diseases.

The purpose of this study was to investigate whether DNA methylation exists in diabetic patients with macrovascular complications and to identify the potential circulating biomarkers for early intervention. This study enrolled 154 participants which were divided into three groups: normal control (NC group, n=43), diabetes without cardiovascular diseases (DM group, n=60), and diabetes with cardiovascular diseases (CVD group, n=51). We performed the methylation gene chip based on DNA microarray combined with methylated DNA immunoprecipitation (MeDIP) method, and then compared abnormal DNA methylated genes between patients with or without cardiovascular diseases. Furthermore, we investigated the expressions of aberrantly methylated genesin the serum of these patients.

## Materials and methods

### Patients and study design

The subjects enrolled in this study were recruited from the endocrinology department of Wuhan Union Hospital from January 2019 to December 2019. This study has been approved by the Institutional Research Ethics Committee of Union Hospital, Huazhong University of Science and Technology. A prior written informed consent was obtained from each participant in this study. The subjects were dividedinto three groups: normal control (NC group, n=43), diabetes without cardiovascular diseases (DM group, n=60), and diabetes with cardiovascular diseases (CVD group, n=51). The inclusion criteria of subjects in the NC group were: no history of diabetes or cardiovascular diseases, normal glucose tolerance in OGTT, normal coronary angiography, no history of carotid artery disease or peripheral arterial disease. Diabetes were diagnosed according to the World Health Organization (WHO, 1999) (18). Data on vital signs, anthropometric factors, medical history and behaviors as well as physical activity were collected. Additional data, including weight, height, BMI, waist circumference, hip circumference, glycemia and glycated hemoglobin were obtained for each individual. Diabetes patients in CVD group were evidenced as follows: ischemic heart disease defined by clinical history, and/or ischemic electrocardiographic alterations; peripheral vascular disease including atherosclerosis obliterans and cerebrovascular disease based on history, physical examinations and Doppler velocimetry.

### MeDIP-chip

Genomic DNA (gDNA) was extracted from nine blood samples (3 from NC group, 3 from DM group, and 3 from CVD group) by a DNeasy Blood & Tissue Kit (Qiagen, Fremont, CA). Using Biomag™ magnetic beads that coupled with the mouse monoclonal antibody against 5-methylcytidine, methylated DNA was immunoprecipitated, which was then eluted and purified by QiagenMinElute columns (Qiagen, Fremont, CA). The total input genomic DNA and immunoprecipitated DNA were labeled with Cy3- and Cy5-fluorophere, respectively. They were then hybridized to NimbleGen Human DNA Methylation 3×720K CpG Island Plus RefSeq Promoter Microarray. Scanning was performed with the Axon GenePix 4000B microarray scanner according to the manufacturer’s guidelines detailed in NimbleGen MeDIP-Chip protocol (NimbleGen Systems Inc., Madison, USA). The MeDIP-chip data were submitted to Gene Expression Omnibus (GEO) data repository (http://www.ncbi.nlm.nih.gov/projects/geo/) under accession number GSE188395.

### Analysis of MeDIP-chip data

The log_2_ ratio obtained from raw data values has been normalized to ensure technical invariability and evaluate methylation differences. Median centering, quantile normalization, and linear smoothing were then performed using Bioconductor packages Ringo, Limma and MEDME (19). We then applied a sliding-window (750bp) peak-finding algorithm which was provided by NimbleScan v2.5 (Roche-NimbleGen) to analysis the MeDIP-chip data. Furthermore, to confirm whether the probes drawnwere from a more positive distribution of intensity log_2_ ratio, a one-sided Kolmogorov-Smirnov (KS) test was applied. Each probe received a -log_10_ P scores from the windowed KS test around that probe. The region of which adjacent probes significantly rose above a set threshold was assigned to an enrichment peak (EP). The peaks were detected by searching for at least two probes that above a P-value minimum cut-off (-log_10_) of 2. Peaks that within 500bp of each other are merged.

### Functional and pathway enrichment analysis

The Gene Ontology (GO) enrichment and Kyoto Encyclopedia of Genes and Genomes (KEGG) pathway analysis were applied to investigate the potential biological processes and the DNA methylation function (20, 21). The DAVID Functional Annotation Tool (http://david.abcc.ncifcrf.gov/) was adopted for GO enrichment, and KEGG pathway analysis with a 0.05 cut-off for Benjamini adjusted *P* value (Q-value) (22). According to GO annotation, the differentially methylated genes were divided into cellular component (CC), molecular function (MF), and biological process (BP). The source genes used to analysis are freely available at https://github.com/hsqhaha/2021-10-08.

### Methylation-specific PCR

In order to confirm the MeDIP-chip data, MSP was applied to identify the methylation status of specific genes (23). Peripheral blood of the subjects (40 from NC group, 57 from DM group, and 48 from CVD group) were collected. The MSP primers are designed by online MethPrimer software (http://www.urogene.org/methprimer). The sequences and product length of each primer used for MSP analysis were listed in **Table 1**.

**Table 1:** Primers of genes for MSP.

Gene name	Type	Sequences(5’ - 3’)	length
VEGFB-Methylated	Forward	ACGGTTAGGGTAGCGGTAGTC	189bp
	Reverse	ACCGAACACGAACTCTACGAA	
VEGFB-Unmethylated	Forward	AATGGTTAGGGTAGTGGTAGTTGT	194bp
	Reverse	AAAAACCAAACACAAACTCTACAAA	
FATP3-Methylated	Forward	ATATTCGGGATAAAATAATTTGTGC	172bp
	Reverse	CCTCACTTCATCTTATAAACTTCGTC	
FATP3-Unmethylated	Forward	ATTTGGGATAAAATAATTTGTGTGT	169bp
	Reverse	CTCACTTCATCTTATAAACTTCATC	
FATP4-Methylated	Forward	AGGTTTTGGAGTTTTTAAAATACGT	201bp
	Reverse	TCAAACAAAAATAAACGAAACGAC	
FATP4-Unmethylated	Forward	AGGTTTTGGAGTTTTTAAAATATGT	203bp
	Reverse	TTCAAACAAAAATAAACAAAACAAC	
PLGF-Methylated	Forward	TTAGAAGATGTTCGAATTATCGGTC	123bp
	Reverse	AAAAACCACCATACTCATCCCC	
PLGF-Unmethylated	Forward	TAGAAGATGTTTGAATTATTGGTTG	122bp
	Reverse	AAAAACCACCATACTCATCCCC	
F2R-Methylated	Forward	AATCGTTTTAGATATAGCGTTCGTC	124bp
	Reverse	TTCACCCTCTCTCCTAACTTCTA	
F2R-Unmethylated	Forward	GTTTTAGATATAGTGTTTGTTGAGG	121bp
	Reverse	CTTCACCCTCTCTCCTAACTTCTAC	
PLCB1-Methylated	Forward	ATTCGTCGCGATTGGTAGTTTC	108bp
	Reverse	GACCATACCCCGAACGAAAC	
PLCB1-Unmethylated	Forward	GAATTTGTTGTGATTGGTAGTTTTG	112bp
	Reverse	CCAACCATACCCCAAACAAAAC	
CAMK1D-Methylated	Forward	TTATCGGTTGTGTGCGTTTATTC	161bp
	Reverse	CCAAAATCTTACCTACACCTCGTAC	
CAMK1D-Unmethylated	Forward	TTATTGGTTGTGTGTGTTTATTTGT	161bp
	Reverse	CCAAAATCTTACCTACACCTCATAC	
DRD5-Methylated	Forward	TAGGTAGTAACGGTATCGCGTATTC	117bp
	Reverse	TAACCACCTATAAAAACCCCAATAA	
DRD5-Unmethylated	Forward	AGGTAGTAATGGTATTGTGTATTTG	117bp
	Reverse	ATAACCACCTATAAAAACCCCAATA	

### Real-time quantitative PCR

qPCR was performed by SYBR Premix Ex Tap™ (Takara, Japan) on the LightCycler^®^ 480 II (Roche Diagnostics Ltd., Basel, Switzerland) following the manufacturer’s instructions. GAPDH was used as the reference genes to normalize the relative mRNA expression. The primer sequences are listed in **Table 2**. The relative quantification analysis was calculated using the 2^-△△Ct^ method (24). The primers of qPCR were designed using the Primer-BLAST program (http://www.ncbi.nlm.nih.gov/tools/primer-blast/) or software packages Primer Premier 5.0.

**Table 2:** Primers of genes for qPCR.

Gene name	Type	Primer Sequence (5’ - 3’)
VEGFB	Forward Reverse	TAATGGGATTTGGGCTTTGG GCACAATGAGGGAGCAAGACA
FATP4	Forward	TCCGCTGGAAAGGTGAGAAC
	Reverse	GCATACAGGGGCAGTTCCTT
PLGF	Forward Reverse	TGTCCAAAGTAGGGATGCG TACAAGCAAATGGCAAAGTG
GAPDH	Forward Reverse	ACTCCTCCACCTTTGACGCTG CTCTCTTCCTCTTGTGCTCTTGC
PLCB1	Forward Reverse	CTCAGCCTTGTCAAAGATGCCA CAAATGGGAGATGTTCACGAGG
DRD5	Forward Reverse	ACTCCTCACTCAACCCCGTC ATGTTCACCGTCTCCACCG

VEGFB, Vascular endothelial growth factor B; FATP4, Fatty acid transport protein 4; PLGF, Placental growth factor; GAPDH, Glyceraldehyde-3-Phosphate Dehydrogenase; PLCB1, Phospholipase C Beta 1; DRD5, Dopamine Receptor D5.

### ELISA

The expressions of PLCB1, DRD5, PLGF, FATP4, and VEGFB in serum were analyzed by enzyme‐linked immunosorbent assay (Blue Gene, Shanghai, China). The measurement was performed following the manufacturer’s instructions. All samples were assessed in triplicate.

### Statistical analysis

Statistical analysis was performed by SPSS 22.0 software and GraphPad 5. The significance was calculated by one-way analysis variance (ANOVA) or Student t-test. All experiments were performed at least three times independently. The results are presented as the mean ± standard error. Differences were statistically significant if P< 0.05.

## Results

### General clinical characteristics

There was no significant difference in age, gender, systolic blood pressure (SBP), and triglycerides (TG) among the three groups (*P*> 0.05) as **Table 3** shown. Compared with NC group, waist-hip ratio (WHR), diastolic blood pressure (DBP), total cholesterol (TC), HbA1c, and fasting plasma glucose (FPG) were significant higher in CVD and DM group (*P*< 0.05). Meanwhile, no obvious difference was observed in those clinical parameters between DM and CVD groups (*P*> 0.05). Consistent with the previous findings, BMI in DM group was significantly higher than that in NC group (*P*< 0.05). Compared with NC group, there was an increased tendency of BMI of diabetic patients in CVD group (*P*> 0.05).

**Table 3:** Clinical characteristics of participants.

	NC	DM	CVD
**Male/female**	24/19	33/27	28/23
**Age (years)**	50.5 ± 8.1	49.3 ± 6.8	52.4 ± 7.9
**BMI (kg/m^2^)**	23.45 ± 1.79	26.39 ± 2.32^*^	24.98 ± 2.21
**WHR**	0.84 ± 0.05	0.96 ± 0.1^*^	0.95 ± 0.06^*^
**SBP (mmHg)**	127.5 ± 9.2	128.1 ± 10.4	133.8 ± 14.6
**DBP (mmHg)**	75.6 ± 6.8	85.4 ± 6.5^*^	88.0 ± 8.7^*^
**TC (mmol/l)**	3.67 ± 0.54	4.24 ± 0.48^*^	4.86 ± 0.67^*^
**TG (mmol/l)**	1.76 ± 1.49	2.2 ± 1.47	2.1 ± 1.53
**HbA1C (%)**	5.1 ± 0.6	7.9 ± 1.2^*^	8.2 ± 1.5^*^
**FPG (mmol/l)**	5.0 ± 0.9	7.5 ± 1.3^*^	7.9 ± 2.4^*^

Data are expressed as means ± SEM, and *P< 0.05, compared to NC group.

SBP, systolic blood pressure; DBP, diastolic blood pressure; WHR, waist-hip ratio; TC, total cholesterol; TG, triglyceride; FPG, fasting plasma glucose.

### DNA methylation status in diabetic patients

The global cytosine methylated peaks in peripheral blood were obtained to assess the DNA methylation variations among the three groups. A total number of 57273 methylated peaks were identified. Among these peaks, 19677 (34.3%) were in NC group, and 20552 (35.9%) methylated peaks were in DM group, and the remaining 17044 (29.8%) methylated peaks were in CVD group.

**Figure 1:**
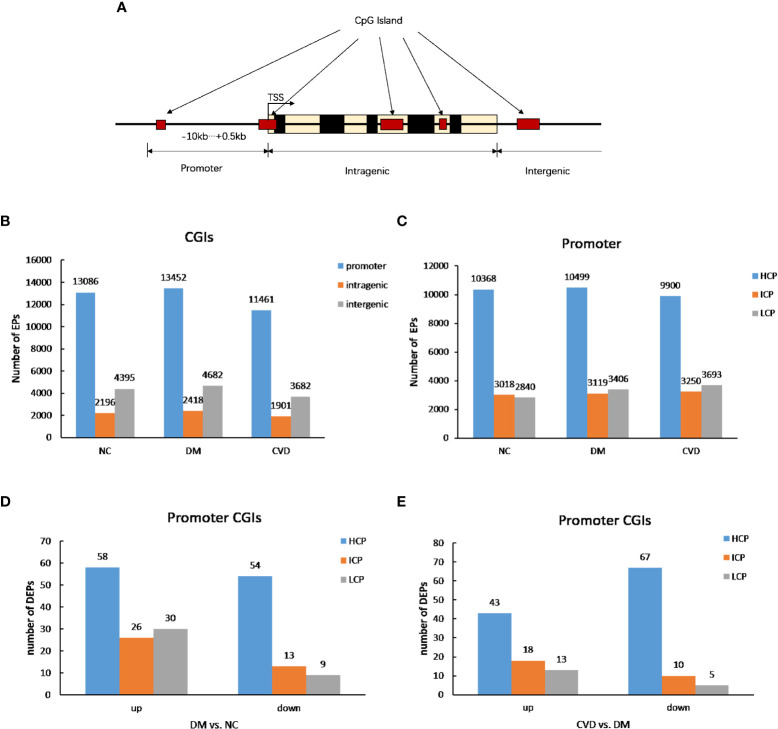
DNA methylation status among different groups. **(A)** Diagram of CpG lands (CGIs) location in gene. CGIs were grouped depending on the distance of sequence which contains high frequency CG sites to RefSeq genes. **(B, C)** Profiles of expression peaks in CGIs and promoter. **(D)** Profiles of different expression peaks between NC (n=3) and DM group (n=3). **(E)** Profiles of different expression peaks between CVD (n=3) and DM group (n=3).

As shown in **Figure 1A**, the CpG lands (CGIs) were grouped into three classes depending on the distance of sequence, which contains high-frequency CG sites to RefSeq genes. They are promoter islands (from about -10kb to +0.5kb around the transcription starting site), intragenic islands (from +0.5kb around the transcription start site to the transcription end site) and intergenic islands (the remainder that does not fall in either promoter or intragenic). The numbers of methylated enrichment peaks (Eps) among different groups were depicted in **Figure 1B**. A number of 13086, 13452, and 11461 methylated EPs were detected in NC, DM, and CVD group, respectively, in promoter CGIs, while the number of EPs in intragenic islands is 2196, 2418 and 1901 in NC, DM and CVD group respectively.

Based on the ratio of CpG, GC contents and the length of CpG-rich region, the promoter regions were subdivided into three classes: high CpG density promoter (HCP), low CpG density promoter (LCP) and intermediate CpG density promoter (ICP). As shown in **Figure 1C**, compared with the number of methylated peaks in ICP and LCP types, HCP type had the highest number among the three groups. Furthermore, both DM and CVD groups have more methylated differential enrichment peaks in HCP than those in ICP and LCP, regardless of the promoters that were either up- or down-methylated (**Figures 1D, E**).

### Differentially methylated genes among CVD, DM, and NC groups

**Figure 2:**
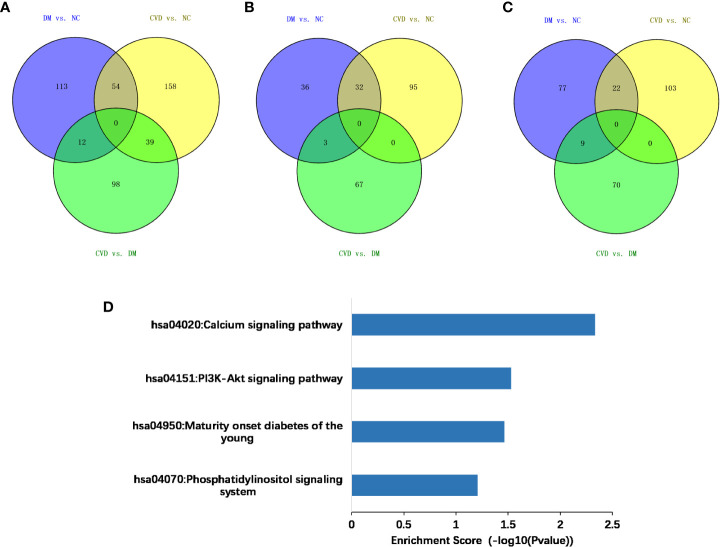
Methylated genes that were unique or overlapped between the DM and CVD group. **(A–C)** Overall different methylated genes, down-regulated genes, and up-regulated genes among three groups, respectively. Purple color represents the result of comparison between DM and NC group. Yellow color represents the result of comparison between CVD and NC group. Green color represents the result of comparison between CVD and DM group. **(D)** Based on DAVID database, KEGG pathway analysis of all aberrant methylated genes shows the high score pathways participating in DM and CVD. The vertical axis is the pathway category; the horizontal axis is the enrichment of pathways.

Compared with the NC group, 179 and 251 genes were methylated in the promoter regions of DM and CVD group, respectively, whereas 54 genes overlapped between these two groups (**Figure 2A**). Of these genes, a total of 211 genes were hypermethylated while 166 were hypomethylated, in which 22 genes and 32 genes were overlapped between DM and CVD group, respectively (**Figures 2B, C**). Meanwhile, the number of methylated genes in CVD group was more than that in DM group, and each group had its special aberrant methylated genes apart from the overlapped genes. Additionally, CVD group had 149 differentially methylated genes compared to the DM, which includes 79 up-regulated genes and 70 down-regulated genes. The above results indicate that DNA methylation modification was widespread in diabetes and its cardiovascular complications. DNA methylation of specific genes might play a role in the outcome of cardiovascular diseases in diabetes.

### Bioinformatics analysis of differentially methylated genes

To analyze the potential role of differentially methylated genes, we performed gene ontology (GO) and pathway enrichment analysis using the DAVID online tool. The threshold was set as*P*< 0.05. In **Figure 2D**, KEGG pathway analysis showed that the calcium signaling pathway and the PI3K-Akt signaling pathway ranked the top 2 pathways according to the enrichment score, which are the critical signaling pathways in the development of DM and its macrovascular complications. GO analysis revealed the functional pathways of abnormally methylated genes in DM and CVD groups, compared with NC group (**Table 4**). Genes participated in the fatty acid metabolic process such as fatty acid transport protein 3 (FATP3) and fatty acid transport protein 4 (FATP4) were screened out to be down-methylated. Meanwhile, genes involved in the vascular endothelial growth factor receptor (VEGFR) signaling pathway such as vascular endothelial growth factor B (VEGFB), placental growth factor (PLGF), and coagulation factor II thrombin receptor (F2R) were found to be down-methylated.

**Table 4:** GO analysis of abnormal methylated genes in DM and CVD compared to NC group.

Category	Term	Pathway	count	P-value
GOTERM_BP_FAT	GO:0031327	negative regulation of cellular biosynthetic process	18	0.036663
GOTERM_BP_FAT	GO:0016055	Wnt receptor signaling pathway	7	0.040088
GOTERM_BP_FAT	GO:0006631	fatty acid metabolic process	10	0.043091
GOTERM_BP_FAT	GO:0009890	negative regulation of biosynthetic process	18	0.043681
GOTERM_BP_FAT	GO:0048010	vascular endothelial growth factor receptor signaling pathway	5	0.048131
GOTERM_BP_FAT	GO:0035176	social behavior	3	0.049028
GOTERM_CC_FAT	GO:0005654	nucleoplasm	27	0.009285
GOTERM_CC_FAT	GO:0043233	organelle lumen	46	0.016427
GOTERM_CC_FAT	GO:0031981	nuclear lumen	38	0.018949
GOTERM_CC_FAT	GO:0031974	membrane-enclosed lumen	46	0.022481
GOTERM_CC_FAT	GO:0070013	intracellular organelle lumen	44	0.026855
GOTERM_CC_FAT	GO:0031410	cytoplasmic vesicle	19	0.043057
GOTERM_MF_FAT	GO:0030528	transcription regulator activity	41	0.028673
GOTERM_MF_FAT	GO:0030552	cAMP binding	3	0.033853
GOTERM_MF_FAT	GO:0031420	alkali metal ion binding	10	0.035444
GOTERM_MF_FAT	GO:0031855	oxytocin receptor binding	2	0.038747
GOTERM_MF_FAT	GO:0031895	V1B vasopressin receptor binding	2	0.038747
GOTERM_MF_FAT	GO:0003700	transcription factor activity	28	0.042173
GOTERM_MF_FAT	GO:0005267	potassium channel activity	7	0.046547

GO, Gene ontology; MF, molecular function; CC, cellular component; BP, biological process. Terms that P value < 0.05 were listed in the table.

### Validation of MeDIP-chip data by MSP

**Figure 3:**
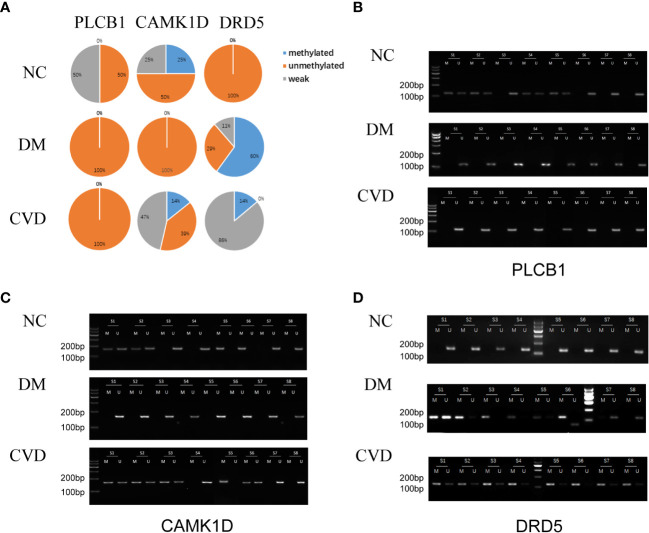
MSP of DNA methylation genes participating in calcium signaling pathway. **(A)** Pie chart shows the distribution of MSP derived from DNA methylation categories in NC (n=40), DM (n=57) and CVD groups (n=48). Weak methylated was defined by methylated band and unmethylated band coming out simultaneously in gel electrophoresis of MSP. **(B–D)** Results of representative MSP analysis at aberrant methylated region of PLCB1, CAMK1D and DRD5 respectively. The location of the amplicon is indicated in **Table 4**. M and U represent the reaction for methylated and unmethylated DNA, respectively.

**Figure 4:**
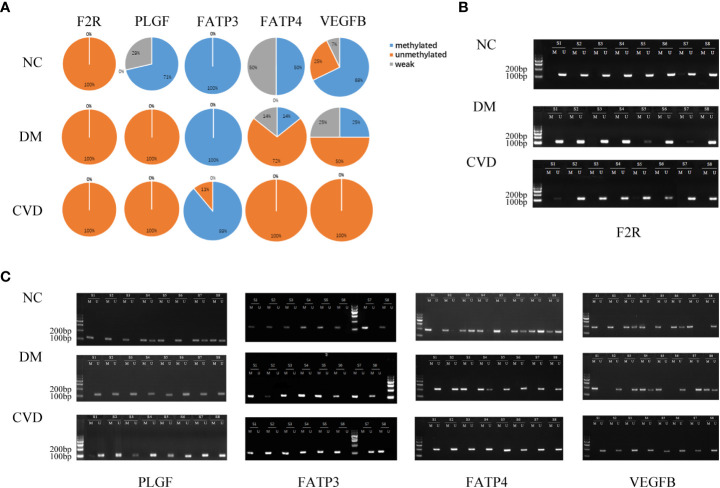
MSP of DNA methylation genes participating in vascular endothelial growth factor receptor signaling pathway. **(A)** Pie chart shows the distribution of MSP derived from DNA methylation categories in NC (n=40), DM (n=57) and CVD groups (n=48). Weak methylated was defined by methylated band and unmethylated band coming out simultaneously in gel electrophoresis of MSP. **(B, C)** Results of representative MSP analysis at aberrant methylated region of F2R, PLGF, FATP3, FATP4 and VEGFB. The location of amplicon is indicated in **Table 4**. M and U represent the reaction for methylated and unmethylated DNA, respectively.

Several studies have demonstrated the important role of calcium signaling pathway in atherosclerosis (25–27). Additionally, dysfunction of vascular endothelial function could enhance atherosclerotic plaque progression (28). Based on the results of bioinformatics analysis, we also found several significantly hypomethylated genes (PLCB1, CAMK1D, and DRD5) participated in the calcium signaling pathway, while several hypomethylated genes (VEGFB, PLGF, F2R, FATP3, and FATP4) participated in VEGFR signaling pathway. Then we performed MSP evaluation using leukocytes from peripheral blood of the subjects. In general, a good correlation between the MeDIP-chip and MSP results was observed for PLCB1, VEGFB, PLGF, and FATP4, except for DRD5. However, MSP of FATP3, CAMK1D, and F2R did not show significant differences among the three groups (**Figures 3**, **4**). Compared with NC group, DM and CVD groups had a significantly lower DNA methylation in PLCB1, VEGFB, PLGF, and FATP4 (*P*< 0.05). Furthermore, the levels of DNA methylation in FATP4 and VEGFB in CVD group were significantly lower than those in DM group (*P*< 0.05). There was no significant difference in PLCB1 and PLGF between DM and CVD group (*P*> 0.05).

### Expressions of differential DNA methylated genes in the serum of CVD, DM, and NC groups

**Figure 5:**
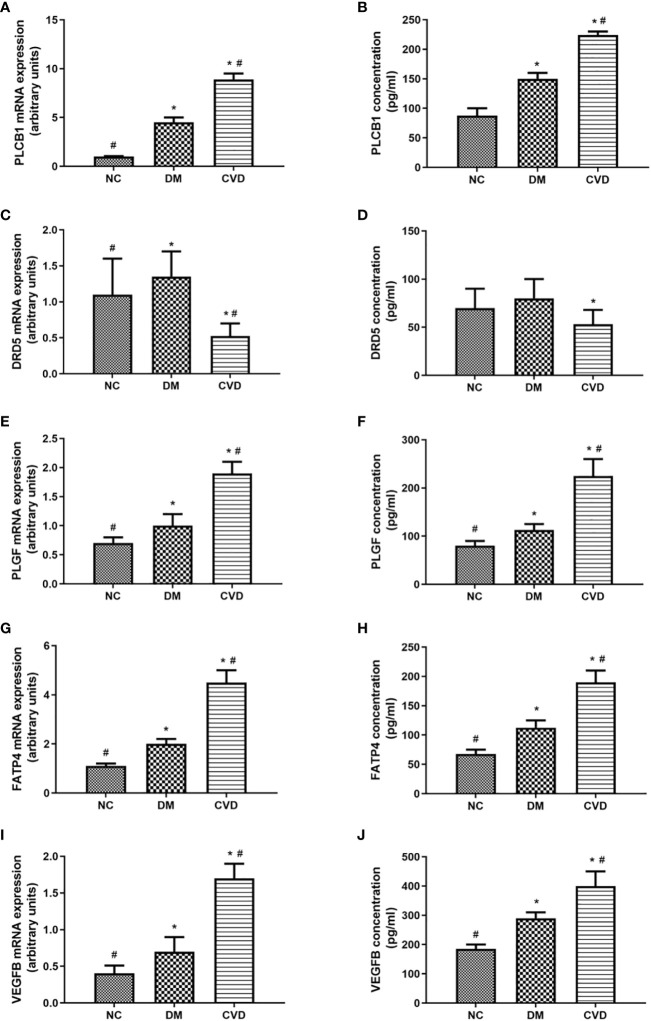
Expressions of differential methylated genes in serum of CVD, DM and NC groups. QPCR of PLCB1 **(A)**, DRD5 **(C)**, PLGF **(E)**, FATP4 **(G)**, VEGFB **(I)** in serum of NC (n=40), DM (n=57) and CVD groups (n=48) respectively. ELISA of PLCB1 **(B)**, DRD5 **(D)**, PLGF **(F)**, FATP4 **(H)**, VEGFB **(J)** in serum of NC (n=40), DM (n=57) and CVD groups (n=48) respectively. Data are presented as mean fold change ± SEM of 3 independent experiments. *P< 0.05, compared to NC group, #*P*< 0.05, compared to DM group.

To further verify the differential methylated genes mentioned above, we performed qPCR to detect the mRNA expression in serum of three groups. The results showed that the levels of *PLGF*, *PLCB1*, *FATP4*, and *VEGFB* in the DM or CVD group were significantly higher than those in NC group (*P* < 0.05) (**Figures 5A, E, G, I**). Elisa results also showed the similar patterns among three groups (**Figures 5A-J**). A good consistency among the DNA methylation, mRNA and protein levels was observed for *PLCB1*, *PLGF*, *FATP4*, and *VEGFB* genes.

## Discussion

Diabetic cardiovascular complications are epidemic and pose a serious threat in modern society. Several studies explored the possible mechanisms involved in the development of diabetes and its associated cardiovascular complications (29–31). However, the precise mechanisms of macrovascular complications in diabetic patients remainto be elucidated. As a widely recognized assay, MeDIP-chip could provide comparative genome-wide DNA methylation dynamic patterns. In this study, MeDIP-chip was conducted to generate the DNA methylation profiles of leukocytes in peripheral blood. Compared to NC group, the level of DNA methylation was higher in DM group, while it was lower in CVD group. The different DNA methylation might be due to the different methylated peaks in the promoter CGIs of the DM and CVD groups. Our results indicate that the low level of DNA methylation of peripheral blood leukocytes may be a biomarker for cardiovascular diseases in diabetic patients. Similarly, Gertrud Lund et al. reported that DNA methylation polymorphisms precede histological signs of atherosclerosis in mice lacking apolipoprotein E (32). Besides, it was also identified that epigenetic modifications were associated with proliferative diabetic retinopathy by genome-wide analysis of DNA methylation in type 1 diabetes patients (33).

Since the differentially methylated regions were mostly enriched in the gene promoter (34), we analyzed the global DNA methylation patterns in the promoter regions. In this study, comparing to the methylation level of intergenic regions, it was significantly higher in the promoter region in human peripheral blood leukocytes. The methylation level of LCP and ICP was lower than that of HCP. In addition, the number of down-methylated CGI promoters was lower than that of up-methylated HCP, LCP, and ICP in DM group, while it was higher than CVD group. These results showed that cardiovascular diseases in diabetes possessed notably methylation changes in CGI promoters of the genome. Similarly, the relationship between CpG methylation pattern of the proximal insulin gene promoter and diabetes had been reported (35, 36). Taking together, we proposed that DNA methylation might play an important role in the pathophysiology of diabetes and its cardiovascular diseases.

In this study, bioinformatics analysis of MeDIP-chip results implied that several signaling pathways might participate in the development of diabetes and its cardiovascular diseases, including calcium signaling pathway and VEGFR signaling pathway. Thus, the validation of MeDIP-chip data by methylation-specific PCR was performed. Furthermore, qPCR and ELISA were conducted to detect the expression of aberrant methylated genes in participants’ serum. The results turned out that only the mRNA and protein expression changes of PLCB1, PLGF, FATP4, and VEGFB were consistent with its methylation status. It is possible that the expression of other aberrant methylated genes might be regulated independent on DNA methylation (37). However, our study indicates that the hypomethylation of VEGFB, PLGF, PLCB1, and FATP4 might upregulate the expression of proteins, respectively. And the level of the proteins might affect the function of vascular endothelial. Several studies have also shown that those gene expressions could be regulated by DNA methylation. Hypomethylated PLCB1 was related to its increased expression in patients with colorectal cancer (38). Hypomethylation of PLGF could increase its expression in lung carcinoma cell lines (39). Folic acid could downregulate the expression of VEGFB by DNA methylation in retinal microvascular endothelial cell (40).

**Figure 6:**
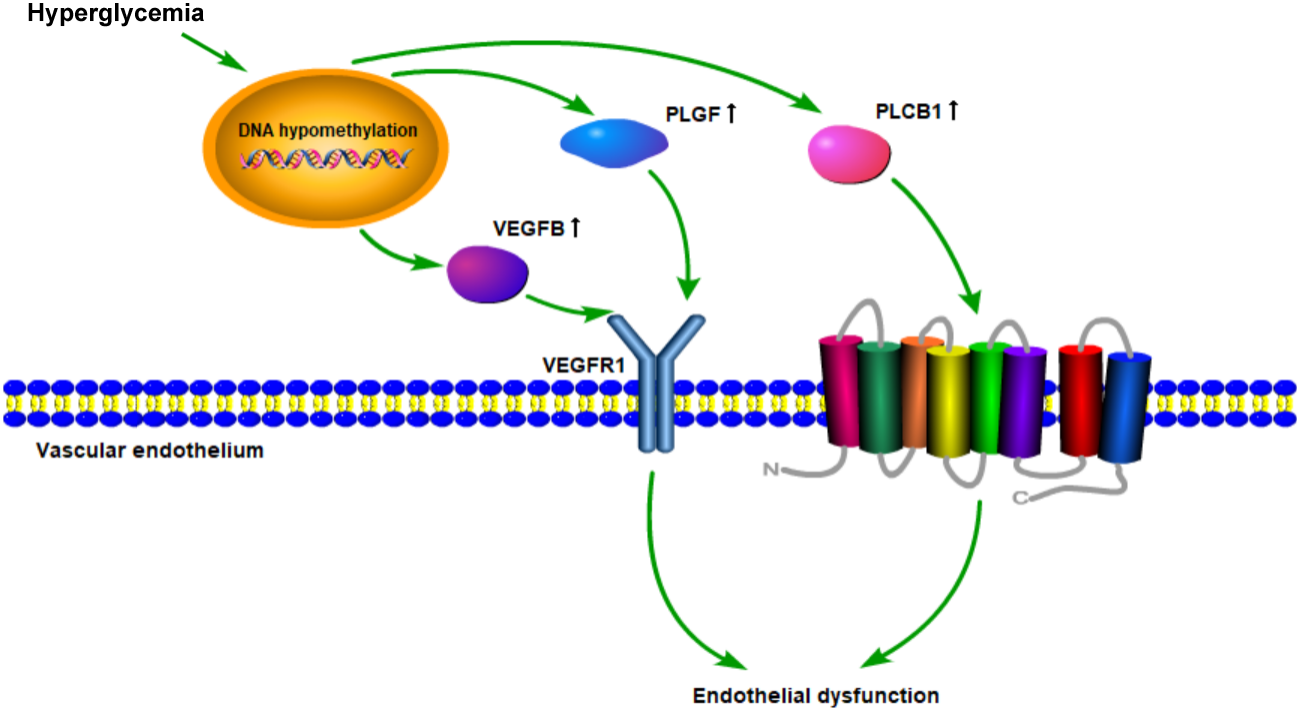
Schematic diagram of the mechanism hypothesis of “VEGFR signaling pathway regulated by DNA methylation in the cardiovascular diseases’ pathogenesis of diabetes”.

The data above also showed that the VEGFR signaling pathway is probably a crucial target of DNA methylation in diabetes patients. Roberto Monastero et al. had demonstrated that the expression of VEGFB gene could be affected by dietary fatty acids (41). This regulation might be mediated, at least in part, by epigenetic modifications on VEGFB promoter methylation. As vascular endothelial growth factor is a key regulator of physiological angiogenesis, which is mediated by binding to its receptors (42), we assume that VEGFR signaling pathway is crucial for diabetic vascular development. The potential hypothesis was shown in **Figure 6**.

Endothelial dysfunction plays a critical role in initiating atherosclerosis (43, 44). It has been demonstrated that VEGFR signaling pathway mainly participates in physiological angiogenesis. Accumulating evidence indicates that intra-plaque angiogenesis promotes atherosclerosis and plaque destabilization (45). Plaque microvascularization and increased endothelial permeability are key players in the development of atherosclerosis, from the initial stages of plaque formation to the occurrence of acute cardiovascular events (46). PLGF and VEGFB could, respectively, bind to VEGFR1, then directly or indirectly inducing angiogenesis (47–49). In addition, as a member of the VEGF family, VEGFB was found to regulate the lipid transport across the vascular endothelium through FATP4 and thus determine lipid accumulation in muscle and heart (50, 51). Moreover, inhibition of VEGFB expression could prevent the progression of type 2 diabetes and restore insulin sensitivity in mice fed a high-fat diet. Our results also demonstrated that DNA methylation of VEGFB was lower in diabetes and its cardiovascular diseases groups than that in the normal group. The expression of VEGFB in peripheral blood leukocytes was correspondingly higher in DM and CVD groups. It has been observed that changes in DNA methylation patterns are associated with coronary artery disease (CAD) (52). Therefore, DNA methylation of genes in VEGFR signaling pathway might play a critical role in potentiating the process of endothelial dysfunction in diabetic cardiovascular diseases.

## Conclusions

This study is the first one to reveal and compare the DNA methylation status of diabetes with or without macrovascular complications. The VEGFR signaling pathway regulated by DNA methylation might participate in the development of diabetic cardiovascular diseases. These findings also showed that the hypomethylation of VEGFB, PLGF, PLCB1, and FATP4 might be the potential biomarkers and therapeutic targets for early interventions in diabetic patients with cardiovascular diseases. The limitation of our study is that our results are obtained in blood DNA. It needs more studies to confirm genes’ function in diabetes and its cardiovascular complications model. In addition, further efforts are required to determine the cause-and-effect relationship between gene methylation and diabetic cardiovascular diseases.

## Data availability statement

The datasets presented in this study can be found in online repositories. The names of the repository/repositories and accession number(s) can be found in the article/supplementary material.

## Ethics statement

The studies involving human participants were reviewed and approved by the Institutional Research Ethics Committee of Union Hospital, Huazhong University of Science and Technology. The patients/participants provided their written informed consent to participate in this study.

## Author contributions

YL and SH conceived the idea and drafted the manuscript. WW, YY, KQ, and YX were responsible for completing experiments. LC and TZ edited the manuscript. All authors contributed to the article and approved the submitted version.
